# Imatinib on target in stroke recovery

**DOI:** 10.1172/JCI190024

**Published:** 2025-03-03

**Authors:** Hae Ryong Kwon, Lorin E. Olson

**Affiliations:** Cardiovascular Biology Research Program, Oklahoma Medical Research Foundation, Oklahoma City, Oklahoma, USA.

## Abstract

Ischemic stroke causes scars in the CNS that impede functional recovery, and there is a need for therapeutics to improve recovery after the acute phase. Scar-resident myofibroblasts and the PDGF pathway have been implicated in stroke pathology. In this issue of the *JCI*, Protzmann et al. report that inhibition of PDGF-CC or its receptor, PDGFRα, reduces the myofibroblast population and improves functional recovery after ischemic stroke in mice. Importantly, PDGFRα inhibition was effective in improving functional recovery even when initiated 24 hours after stroke, which suggests opportunities for later treatment by targeting the PDGF pathway. This study demonstrates the therapeutic potential of enhancing stroke recovery even after acute damage and blood-brain barrier dysfunction has already occurred.

## Expanding the window of time for stroke treatment

Ischemic stroke is a major cause of death and disability, with few treatment options. One treatment is intravenous tissue plasminogen activator (tPA), which can promote thrombolysis early after a stroke. However, tPA proteolytically activates PDGF-CC, one of the ligands for PDGFRα, which leads to blood-brain barrier (BBB) damage (1). BBB dysfunction activates cells in the neurovascular unit (NVU), leading to leukocyte infiltration and the formation of a scar in the following days and weeks. Thus, neuroprotective strategies targeting the BBB are an area of ongoing research. In preclinical studies, inhibition of PDGF signaling with imatinib, a tyrosine kinase inhibitor, can reduce BBB leakage and improve neurological outcomes in mice (1) and humans (2). However, the therapeutic window for stroke treatments is narrow, and imatinib’s efficacy may depend on prompt administration.

PDGFRα is a member of the type 3 receptor tyrosine kinase family, which includes PDGFRβ, Kit, CSF1R, and FLT3. Ligand binding to these receptors leads to dimerization and autophosphorylation on multiple tyrosine residues in the cytoplasmic domain, which triggers recruitment and activation of various signaling proteins (3). There are four genes encoding secreted PDGF ligands that occur as four homodimers: PDGF-AA, PDGF-BB, PDGF-CC, and PDGF-DD. There are also PDGF-AB heterodimers. Depending on the ligand, PDGFRα or PDGFRβ homodimers can form, as well as PDGFRα:PDGFRβ heterodimers. For PDGF-CC, the receptor complex always includes PDGFRα, such that it activates PDGFRα:PDGFRα homodimers and PDGFRα:PDGFRβ heterodimers. PDGF promotes scar formation by inducing cell migration, proliferation, and extracellular matrix (ECM) production. In the circulation, PDGF is found mostly in platelet α-granules in the form of PDGFs AA, BB, and AB, which are released during clot formation along with TGF-β. In contrast, PDGF-CC is predominantly secreted by cells in the tissue itself, where it binds to ECM and awaits proteolytic activation. The precise role of PDGF signaling in the CNS injury response and subsequent scarring is still unclear. PDGFR inhibition reduced scar formation in the context of traumatic brain injury in rodents and stroke injury in rodents and humans (1, 2, 4). In PDGFRβ-heterozygous mice, scarring was reduced but infarct volume was increased in a stroke model (5). On the other hand, PDGFRβ is known to be critical for vascular development (6) and beneficial for BBB integrity (7). Some PDGF signaling is likely to help in stroke recovery, but too much is detrimental.

**Figure 1:**
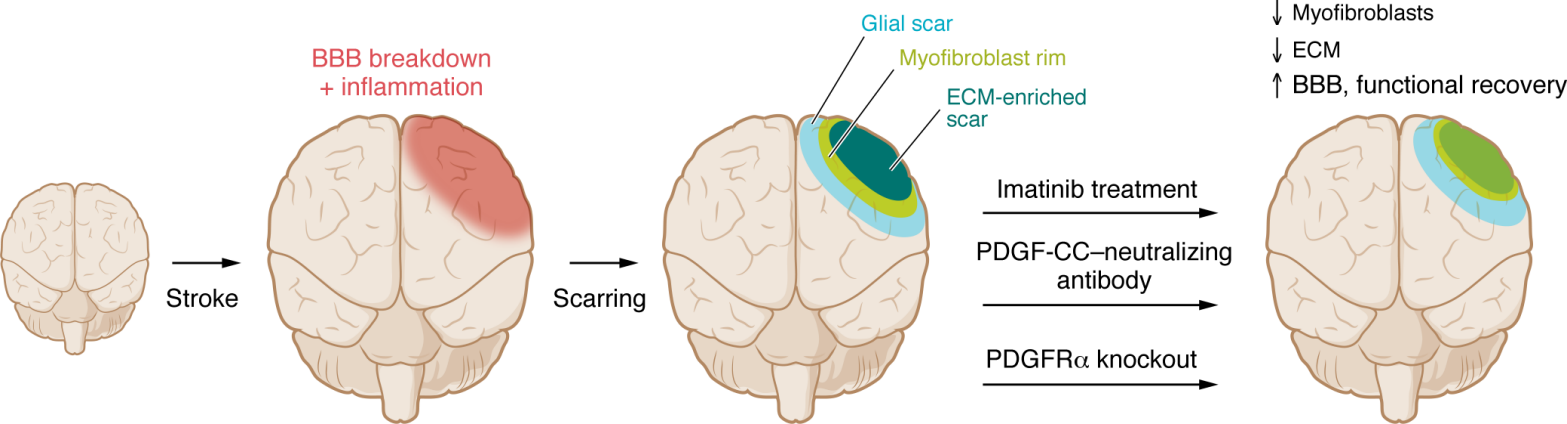
Protzmann et al. demonstrate benefits of PDGF-CC/PDGFRα pathway inhibition for stroke recovery. Experimental stroke was induced using middle artery occlusion in mice, resulting in immediate ischemia, rapid BBB breakdown, and inflammation. Within a few days, a multilayered scar formed with an ECM-rich core, a rim of PDGFRα^+^ myofibroblasts, and an outermost glial layer. Imatinib blocked PDGFRα tyrosine kinase activity. This treatment reduced myofibroblasts and ECM in the scar, and improved BBB integrity and function in sensory-motor integration tests. Similar results were obtained from specifically blocking PDGF-CC with neutralizing antibody, or from genetic deletion of PDGFRα.

In this issue of the *JCI,* Protzmann et al. (8) sought to better understand PDGF signaling mechanisms and how they relate to early and late pathogenic events in ischemic stroke. Using a laser-activated chemical agent to induce controlled ischemic stroke in mouse models via middle cerebral artery occlusion, the authors found that pretreatment with imatinib could block vascular leakage in the acute period after stroke, which coincided with preservation of NVU organization (8). Reactive gliosis is a rapid response to CNS injury that initiates scar formation through the expansion of astrocytes, glial cells, and microglia in the damaged area, and imatinib dampened this early response (8). A few days after stroke, a multilayered scar forms in the damaged area. The scar is composed of an outer glial layer of astrocytes and oligodendrocyte progenitor cells surrounding a fibrotic core of non-neural fibroblasts that secrete collagen and may transition into myofibroblasts (Figure 1). In their stroke model, at 7 days after injury, the authors characterized a layer of PDGFRα^+^ myofibroblasts between the glial layer and the fibrotic core that was reduced by imatinib treatment. Interestingly, inhibition of myofibroblasts was specific, as the glial portion of the scar was not changed. Damage to one side of the brain causes ipsilateral bias when animals interact with their environment. Imatinib reduced this bias and improved sensory-motor recovery at 3 and 7 days after injury. These studies were all performed in animals pretreated with imatinib, but what benefits might carry over into a clinical scenario where treatment would occur after injury? To test this, the authors administered imatinib to mice 24 hours after artery occlusion, when BBB damage had already occurred. They found that posttreatment administration reduced fibrotic scar expansion to the same extent as pretreatment administration, thus indicating that the fibrotic scar formation was independent of early BBB leakage. Furthermore, while only pretreated animals showed functional recovery at 3 days after injury, both treatment groups showed recovery at 7 days. This portion of the study suggests that late intervention with PDGF inhibitors might improve functional recovery by targeting the myofibroblastic component of the CNS scar (8).

## Imatinib on target

What do we learn about PDGF signaling in ischemic stroke? Imatinib inhibits other tyrosine kinases besides PDGFRα, including PDGFRβ, Kit, and Abl. Notably, Protzmann and colleagues found that in untreated animals, PDGFRβ was coexpressed with PDGFRα in myofibroblasts (8). They also identified heterogeneity in this population, with high PDGFRα and PDGFRβ coexpression in the fibrotic cells closest to the glial scar layer and lower PDGFRα expression on PDGFRβ^+^ cells closer to the core. To test the specificity of PDGFRα in their model, the authors used GFAP-Cre, which is selective for astrocytes, to genetically delete *Pdgfra*. As in the imatinib treatment experiments, specific deletion of *Pdgfra* reduced the myofibroblast scar without affecting the astroglial scar. This experiment needs careful interpretation, because GFAP-Cre is active during development, it may not be perfectly specific to astrocytes, and its expression may transiently expand beyond glial cells following injury. However, the experimental result clearly indicates that reducing PDGFRα activity is enough to block myofibroblast expansion and suggests that imatinib may work similarly. The group also used a neutralizing antibody to block PDGF-CC, which exclusively signals through PDGFRα, and this also selectively reduced the myofibroblast scar. Thus, the PDGF-CC/PDGFRα signaling pathway is required for myofibroblast expansion in CNS scar formation, and PDGFRα is the probably the most relevant target for imatinib in this setting.

## Existential questions about scars

Scarring is an adaptive response that is fundamental to the organism’s natural injury response, but it has downsides for functional recovery. Scarring typically occurs at the expense of regeneration, and this is true in most organs as well as the CNS. For example, rapid scar formation in the skin and heart works as a form of patch repair to keep the organ intact after wounding or infarction, but the scarred skin does not sweat or grow hair, and scarred heart does not contract like cardiac muscle. In the brain and spinal cord, scar formation inhibits axon regrowth. The nervous system builds a unique type of scar with an outer glial layer that limits the spread of inflammation and cell death and an inner core of fibroblasts and immune cells that provide structural support and debris clearance, respectively. When they occur, scar fibroblasts with α–smooth muscle actin (α-SMA) expression are called myofibroblasts because of their muscle-like gene expression and contractile function of pulling the surrounding tissue together and lessening the size of the scar. Myofibroblast-generated contraction in nervous system scars might help compress the scar to reduce its volume and avoid cavitation. Hence, controlling myofibroblasts is probably a better goal than eliminating them completely.

The origin of myofibroblasts in different organs has been a topic of much discussion (9, 10). Many cell types have the potential to express myofibroblast genes under experimental conditions (e.g., cells treated with TGF-β or grown on rigid plastic), but lineage tracing, when properly controlled, has consistently identified cells of mesenchymal origin as the major source of myofibroblasts in vivo. In the CNS, mesenchymal cell types can be classified as mural cells (vascular smooth muscle cells and pericytes) and fibroblasts. Both cell types are concentrated in the perivasculature and are themselves heterogeneous and subclassifiable, as shown by single-cell sequencing and lineage tracing (11, 12). CNS injury models with lineage tracing, single-cell RNA sequencing, and spatial transcriptomics have identified the heterogeneity of fibroblasts contributing to scar formation (13, 14). Interestingly, perivascular fibroblasts express PDGFRα and PDGFRβ, but mural cells express only PDGFRβ, and this is true in most organs (11). In spinal cord injury, collagen Ia1–expressing perivascular fibroblasts were suggested as a main source of fibrotic scar formation (15). Perivascular fibroblasts in the CNS are not well understood, but their origins in the developing meninges and migration into the CNS along vascular tracts have been shown recently (16). The finding by Protzmann et al. that myofibroblasts were reduced by early PDGF-CC inhibition supports the interpretation that perivascular fibroblasts were the source of myofibroblasts in their model (8). The observation of myofibroblast heterogeneity in terms of PDGFRα and PDGFRβ coexpression could be suggestive of distinct myofibroblast progenitors, but it is equally consistent with a single progenitor undergoing diversification during scar evolution.

## Future studies

The study by Protzmann et al. points to the next steps for future research and raises several important questions (8). The possibility that imatinib could be effective in stroke recovery when given in the post-acute period obviously demands further, expanded investigation. Key questions about the cellular identity of PDGFRα^+^ cells need to be resolved. In the current study, PDGFRα^+^ perivascular cells coexpressed GFAP and AQP4, which raises questions about whether they were fibroblasts that transiently expressed astrocyte genes or if astrocytes transiently expressed PDGFRα, because fibroblasts and astrocytes in healthy conditions do not share these markers. Time-dependent heterogeneity of PDGFRα^+^ cells suggests different cellular responses to treatment (8). This possibility can be explored by single-cell analysis and lineage tracing with cell-specific Cre strains and intravital microscopy. Lineage barcoding with CRISPR may help to clarify clonal involvement of heterogeneous fibroblasts (17). Protzmann et al. reported that PDGFRα inhibition did not change glial scar formation, but it is unclear whether and how astroglia were affected at the cellular and molecular level (8). Since the glial scar is proposed to have beneficial and detrimental roles, it will be important to know more about how PDGFRα inhibition affects glial scarring and axon regrowth.

## Version 1 03/03/2025

Electronic publication
